# New insight in immunotherapy and combine therapy in colorectal cancer

**DOI:** 10.3389/fcell.2024.1453630

**Published:** 2025-01-07

**Authors:** Kai Ji, Hang Jia, Zixuan Liu, Guanyu Yu, Rongbo Wen, Tianshuai Zhang, Zhiying Peng, Wenjiang Man, Yucheng Tian, Can Wang, Qianlong Ling, Wei Zhang, Leqi Zhou, Mulin Liu, Bing Zhu

**Affiliations:** ^1^ Department of Gastrointestinal Surgery, The First Affiliated Hospital of Bengbu Medical University, Bengbu, Anhui, China; ^2^ Department of Colorectal Surgery, Shanghai Changhai Hospital, Naval Medical University, Shanghai, China

**Keywords:** immunomodulatory, ICI_S_, radiotherapy, chemotherapy, combination therapy, colorectal cancer

## Abstract

The advent of immune checkpoint inhibitors (ICIs) in colorectal cancer (CRC) treatment marks a major breakthrough. These therapies have proven safer and more effective than traditional radiotherapy and targeted treatments. Immunotherapies like pembrolizumab, nivolumab, and ipilimumab have pioneered new treatment avenues, potentially improving patient outcomes and quality of life. Additionally, advances in immunotherapy have prompted detailed research into CRC therapies, especially those integrating ICIs with conventional treatments, providing new hope for patients and shaping future research and practice. This review delves into the mechanisms of various ICIs and evaluates their therapeutic potential when combined with radiotherapy, chemotherapy, and targeted therapies in clinical settings. It also sheds light on the current application and research involving ICIs in CRC treatment.

## 1 Introduction

Colorectal cancer is a prevalent gastrointestinal malignancy, ranking as the third most common cancer globally and the second leading cause of cancer-related deaths (Zhao et al., 2022a). According to “Cancer Statistics 2023,” colorectal cancer incidence rates are similar across genders in the United States, but mortality rates are higher in men than in women (Siegel et al., 2023). Despite recent advancements in surgery, radiotherapy, chemotherapy, and targeted therapy, the overall effectiveness of these treatments for CRC remains suboptimal. ICIs represent a novel immunotherapeutic approach that enhances immune responses and modifies the microenvironment, improving survival outcomes for CRC patients (Tang et al., 2021; Lin et al., 2020). As a leading nation in gastrointestinal malignancies, China often identifies these conditions at intermediate or advanced stages. The primary treatment involves surgery supported by adjuvant radiotherapy and chemotherapy, key for extending patient survival. Despite some successes, major challenges persist, including high postoperative recurrence rates and lower 5-year survival rates (Biller and Schrag, 2021). Previously, treatment for advanced colorectal cancer was confined to conventional chemotherapy and targeted therapy, offering limited options and often suboptimal outcomes. In cases of metastatic colorectal cancer (mCRC), while systemic chemotherapy has improved survival rates, the risk of disease recurrence remains for some patients. Furthermore, the systemic toxicity and limited selectivity of traditional treatments highlight the urgent need for new therapeutic approaches in CRC. Recently, immunotherapy has emerged as a compelling option, becoming a primary treatment modality for metastatic or recurrent tumors (Carlino et al., 2021; Wang et al., 2023a). The introduction of immunotherapy has brought renewed hope to CRC patients, especially the 15% with mismatch repair deficiencies and high microsatellite instability (d-MMR/MSI-H), providing them with more effective and comprehensive treatment option (Ganesh et al., 2019; Li et al., 2023a). The advantages of combining immunotherapy with traditional treatments are clear, yet optimal strategies remain to be determined. Ongoing research in this area will shape future treatment protocols, particularly in creating predictive biomarkers and personalized treatment plans (Silva et al., 2022; Zhang et al., 2022). This review offers a detailed analysis of CRC characteristics and recent research developments, providing insights into the mechanisms of relevant ICIs, current combination therapies, and emerging therapeutic strategies.

## 2 Mechanisms of ICIs in colorectal cancer immunotherapy

Immune checkpoints act as a natural regulatory mechanism within the immune system and are extensively researched in cancer therapy. They help maintain immune balance and prevent excessive activation, which could lead to tissue damage (Carlino et al., 2021). Tumor cells amplify immune regulatory ligands to inhibit the anti-tumor activities of T cells. ICIs counteract these signals, enhancing anti-cancer responses that lead to the death of cancer cells (Sedano et al., 2022; Liu et al., 2021a). In 2011, the FDA approved the anti-CTLA-4 antibody ipilimumab for melanoma treatment, marking the beginning of a new era in clinical therapy. ICIs enhance anti-tumor responses by reducing T cell suppression, specifically targeting CTLA-4 and PD-1 pathways to increase their efficacy against tumors (Naimi et al., 2022). CTLA-4 is an inhibitory receptor on T cells that limits early activation by interacting with CD80/CD86. The use of anti-CTLA-4 antibodies or reduction in CTLA-4 function increases CD28 ligand availability, which activates autoreactive T cells, modulates regulatory T cell (Tregs) homeostasis, and impacts immune responses (Hosseini et al., 2020; Rowshanravan et al., 2018). The interaction between PD-1 and its ligands, PD-L1/L2, is crucial for maintaining T cell tolerance and facilitating tumor immune suppression. This interaction significantly impairs T cell signaling, reduces their functional activity, and decreases cytokine production and cytotoxicity (Chi et al., 2021; Parvez et al., 2023).

In addition to targeting CTLA-4 and PD-1/PD-L1, ICIs also influence other regulatory pathways such as TIM-3, LAG-3, and TIGIT, which are involved in immune cell exhaustion and suppression. Targeting these molecules can enhance the efficacy of immunotherapy (Zhao et al., 2021; Chocarro et al., 2021; Chauvin and Zarour, 2020; Cai et al., 2023). The immunosuppressive characteristics of the tumor microenvironment can hinder immune cell attacks on tumors, thereby diminishing the efficacy of immunotherapy (Bader et al., 2020). The efficacy of ICIs can be influenced by factors such as tumor immunogenicity and the presence of immune cells like myeloid-derived suppressor cells (MDSCs), Tumor-associated Macrophages (TAMs), and Tregs. Therefore, comprehensive activation of the immune system is crucial for effectively combating tumors (Chen et al., 2021; Xue et al., 2021). Research shows that tumors with high immunogenicity or those within an inflammatory microenvironment have better responses to ICIs, while non-immunogenic tumors exhibit poorer treatment outcomes. Therefore, understanding the immune characteristics of the tumor microenvironment and the effects of ICIs is crucial for improving treatment strategies (Kwapisz, 2021; Escobar et al., 2023). Recent studies demonstrate that immunogenic chemotherapy enhances the efficacy of ICIs by facilitating the infiltration of CD4^+^ and CD8^+^ T cells into tumors and reducing the activity of immunosuppressive cells such as Tregs and MDSCs (Pfirschke et al., 2016). Current research focuses on employing a combination of various ICIs and integrating immunotherapy with chemotherapy, radiotherapy, and targeted therapy. This comprehensive approach aims to enhance immune system stimulation and improve the efficacy of cancer treatments (Rotte, 2019; Ban et al., 2021; Ferrara et al., 2020; Spurr et al., 2022). In cancers that are resistant to both conventional chemotherapy and immunotherapy, combining ICIs with systemic chemotherapy significantly improves tumor response compared to ICI monotherapy (Zhao et al., 2022b). Additionally, this combination approach is expected to reduce drug resistance and decrease the toxic side effects associated with individual therapies, thereby improving treatment outcomes and enhancing patients’ quality of life.

While traditional treatments such as surgery, radiotherapy, and chemotherapy provide therapeutic benefits for CRC, their effectiveness is limited in advanced or metastatic stages. Traditional chemotherapy has long been the standard treatment for mCRC; however, its limitations, including systemic toxicity, low response rates, and suboptimal efficacy, highlight the urgent need for more tumor-specific therapeutic strategies (van der Stok et al., 2017). On the other hand, immunotherapy, especially with the use of ICIs, has shown great promise in these scenarios. Clinical trials have demonstrated significant improvements in survival rates and lifespans for patients with advanced or mCRC (Ganesh et al., 2019; Feng et al., 2021; Khalil et al., 2016). For instance, 307 untreated patients with mCRC characterized by high microsatellite instability or mismatch repair deficiency were randomly assigned to receive either pembrolizumab or chemotherapy, with a median follow-up period of 32.4 months. After 18 weeks of treatment, the median time to deterioration in GHS/QOL, physical function, social function, and fatigue scores was significantly longer in the pembrolizumab group compared to the chemotherapy group (Andre et al., 2021). Studies have demonstrated that, compared to chemotherapy, pembrolizumab provides durable antitumor activity and induces fewer treatment-related adverse events, supporting its role as an effective first-line therapy for patients with mCRC characterized by high microsatellite instability or mismatch repair deficiency (Diaz et al., 2022). The deployment of CTLA-4 and PD-1 inhibitors, whether used singly or in combination, represents a significant breakthrough in oncology (Im and Pavletic, 2017; Zhang et al., 2021). The NICHE trial reported that 60% of dMMR CRC patients achieved a pathologic complete response (pCR) following a neoadjuvant immunotherapy regimen with nivolumab and ipilimumab (Chalabi et al., 2020).

CRC patients are categorized into three groups based on their mutation profiles: microsatellite instability-high (MSI-H), microsatellite instability-low (MSI-L), and microsatellite stable (MSS) (Taieb et al., 2022). ICIs show limited efficacy in MSS-CRC, yet demonstrate improved performance in MSI-CRC(43). The enhanced effectiveness of treatments for MSI-CRC tumors is likely due to their high mutational burden, a greater number of neoantigens, and increased immune cell infiltration (Zhao et al., 2022b; Johdi and Sukor, 2020). About 15% of CRC cases show microsatellite instability (MSI), which stems from faulty DNA mismatch repair mechanisms. In more than 75% of sporadic cases, MSI results from the epigenetic silencing of the MLH1 gene. In other cases, it is linked to Lynch syndrome due to inherited mutations in mismatch repair genes (MLH1, MSH2, MSH6, PMS2) (Taieb et al., 2022; Boland and Goel, 2010). Notably, microsatellite instability is a positive prognostic factor in CRC and closely correlates with the presence of intratumoral T cells (Bai et al., 2021). While most MSI tumors respond well to ICIs, the majority of MSS tumors show significant resistance to these agents (Lin et al., 2023). Reports suggest that combining multi-pathway approaches with PD-1/PD-L1 inhibitors improves the effectiveness of anti-PD-1/PD-L1 therapy in MSS CRC by increasing CD8^+^ T cell counts, upregulating PD-L1 expression, and enhancing the tumor microenvironment (Cai et al., 2024). Studies have emphasized the synergistic effects of combining regorafenib with ICIs, presenting promising therapeutic options for patients with MSS-CRC (Akin Telli et al., 2022). The FDA has approved pembrolizumab and nivolumab for treating patients with MSI-H CRC. Furthermore, ipilimumab, in combination with nivolumab, has been approved for MSI-H CRC patients following chemotherapy, demonstrating higher remission rates and significant clinical benefits (Morse et al., 2020). Research shows that blocking IL-17A enhances the effectiveness of anti-PD-1 therapy in MSS CRC mouse models, making it a viable therapeutic target to improve ICI treatment response in MSS CRC patients (Liu et al., 2021b). Concurrently, the METIMMOX trial (NCT03388190) investigating oxaliplatin-based chemotherapy (FLOX) combined with nivolumab versus FLOX alone for MSS mCRC did not meet its primary objectives, highlighting the necessity for further research (Ree et al., 2024; Meltzer et al., 2022). While MSI status is an established biomarker for predicting immune therapy responses, some reports indicate that certain MSS-CRC patients also benefit from ICI treatment (Fabrizio et al., 2018). Consequently, identifying more precise and reliable predictive factors is essential. Lymphocyte Activation Gene 3 (LAG-3) is an emerging immune checkpoint that triggers immune cell apoptosis and decreases cytokine secretion, making it a promising research target for mCRC. Additionally, tumor mutational burden (TMB) serves as another predictive factor for immunotherapy in MSS CRC (Fan et al., 2021). Higher TMB levels, which produce a greater quantity of neoantigens, are associated with increased immunogenicity and improved immunotherapy outcomes, including higher objective response rate (ORR) and longer median progression-free survival (Marabelle et al., 2020). Research trials have shown that combining Favezelimab and pembrolizumab exhibits effective anti-tumor activity, particularly in individuals with a Combined Positive Score (CPS) for PD-L1 ≥ 1 (Garralda et al., 2022). Despite the proven efficacy of combination immunotherapy, continuous exploration is crucial for future clinical applications. Additionally, chimeric antigen receptor (CAR)-T cell therapy and cancer vaccines are active research areas in modern CRC treatment. Although these modalities are still investigational, they have shown promising results in clinical trials (Johdi and Sukor, 2020; Chen et al., 2023).

## 3 Advantages of immune checkpoint inhibitors in colorectal cancer

ICIs exhibit a wide range of anti-cancer activities and have shown durable and potent effectiveness in select cancer patients. To date, over ten PD-1/PD-L1 inhibitors and various other ICIs have received approval for cancer therapy (Table 1).

**TABLE 1:** List of approved immune checkpoint inhibitor.

Medication	Target	Regulatory agency/Region	Time to market	Specification	Indications
Pembrolizumab	PD-1	FDA, EMA, NMPA, Health Canada, TGA	2014.9	100 mg	HL, Melanoma, NSCLC, SCLC, RCC, HNSCC, BC, CRC, HCC, EC^2^, GC, TNBC
Nivolumab		FDA, EMA, NMPA, Health Canada, TGA	2014.12	40/100 mg	Melanoma, NSCLC, SCLC, RCC, HL, HNSCC, BC, CRC, HCC, EC^2^, GC
Camrelizumab		NMPA	2019.5	200 mg	HL, NSCLC, HCC, EC^2^, NPC
Toripalimab		NMPA	2018.12	80/240 mg	Melanoma, NPC, BC, EC^2^, NSCLC
Sintilimab		NMPA	2018.12	100 mg	HL, NSCLC, EC^2^, HCC, EGJA
Tislelizumab		FDA,NMPA	2019.12	100 mg	HL, BC, NSCLC, HCC, NPC, EC^2^, MSI-H/dMMR ST, EGJA
Zimberelimab		NMPA	2021.8	120 mg	HL, CC
Penpulimab		NMPA	2021.8	100 mg	HL,NSCLC
Serplulimab		NMPA	2022.3	100 mg	MSI-H/dMMR ST, NSCLC
Pucotenlimab		NMPA	2022.7	100 mg	MSI-H/dMMR ST
Dostarlimab		FDA, EC^1^, Health Canada	2021.4	500 mg	EC^3^
Cemiplimab		FDA, EC^1^, Health Canada, TGA	2018.9	350 mg	BCC, NSCLC, CSCC
Retifanlimab		FDA, Health Canada	2023.3	500 mg	MCC, SCAC
Sugemalimab	PD-L1	NMPA	2021.12	600 mg	NSCLC
Envafolimab		NMPA	2021.11	200 mg	MSI-H/dMMR ST
Adebrelimab		NMPA	2023.3	600 mg	SCLC
Atezolizumab		FDA, EMA, NMPA, TGA	2016.5	1,200 mg	NSCLC, BC, TNBC, RCC, HCC, GC
Durvalumab		FDA, EMA, NMPA, TGA	2017.3	120 mg/500 mg	NSCLC, SCLC, BC
Avelumab		FDA, EMA, NMPA, TGA	2017.5	200 mg	Melanoma, BC, MCC, RCC
Ipilimumab	CTLA-4	FDA, EMA, NMPA, TGA	2011.3	50 mg/200 mg	MPM, melanoma, CRC, RCC, NSCLC
Tremelimumab		FDA	2022.4	25 mg/300 mg	NSCLC, UHCC
Candonilimab	PD-1/CTLA-4	NMPA	2022.6	125 mg	CC
Relatlimab	LAG-3	FDA	2022.3	80 mg	Metastatic melanoma

EMA, European Medicines Agency’s; NMPA, National Medical Products Administration; EC^1^, European Commission; TGA, Therapeutic Goods Administration; HL, Hodgkin’s lymphoma; NSCLC, Non-Small Cell Lung Cancer; SCLC, Small cell lung cancer; RCC, Renal cell carcinoma; CC, Cervical Cancer; HNSCC, Head and neck squamous cell carcinomas; MCC, Merkel Cell Carcinoma; BCC, Basal Cell Carcinoma; UHCC, Unresectable Hepatocellular Carcinoma; BC, Bladder Cancer; HCC, Hepatocellular Carcinoma; EC^2^, Esophageal Cancer; GC, Gastric Cancer; EGJA, Esophagogastric Junctional Adenocarcinoma; CSCC, Cutaneous Squamous Cell Carcinoma; MPM, Malignant Pleural Mesothelioma; TNBC, Triple-Negative Breast Cancer; NPC, Nasopharyngeal Carcinoma; SCAC, Squamous Cell Anal Carcinoma; EC^3^, Endometrial Cancer; MSI-H/dMMR ST, Microsatellite Instability-High/Deficient Mismatch Repair Solid Tumor.

### 3.1 PD-1/PDL-1 and CTLA-4 inhibitors

PD-1, a key immune checkpoint molecule, inhibits T cell activation and cytokine production by binding to its ligands PDL1/PDL2, thereby preventing peripheral immune system responses and hyperactivation (Parvez et al., 2023). Monoclonal antibodies targeting PD-1 and PD-L1 have been developed to disrupt this immunosuppressive pathway and trigger T cell responses against tumor cells. In MSS mCRC, the ORR were 40% for mismatch repair-deficient tumors and 0% for mismatch repair-proficient tumors, highlighting the higher sensitivity of mismatch repair-deficient tumors to PD-1 blockade (Le et al., 2015). Pembrolizumab shows superior progression-free survival (PFS), a lower incidence of adverse events, and more lasting anti-tumor effects in MSI-CRC patients compared to chemotherapy, although there is no significant difference in overall survival (OS) between the treatment groups (Diaz et al., 2022). To further evaluate the efficacy and safety of the KEYNOTE-177 study, an analysis of health-related quality of life (HRQOL) showed clinically significant improvements in MSI-H CRC patients treated with pembrolizumab compared to chemotherapy. These findings support the use of pembrolizumab as a first-line treatment for this group (Andre et al., 2021). Nivolumab monotherapy achieved an ORR of 31.1% and a disease control rate (DCR) of 69% in previously treated patients (Overman et al., 2017). In the CheckMate 142 Phase II trial, the combination of Nivolumab and Ipilimumab was evaluated for efficacy and safety in MSI-mCRC patients, showing an ORR of 69% and a disease control rate (DCR) of 84%, with a complete response rate (CR) of 13% (Lenz et al., 2022a). Nivolumab combined with low-dose ipilimumab showed robust and durable clinical benefits and was well tolerated as a first-line treatment for MSI-H mCRC (Lenz et al., 2022b). These findings support pembrolizumab as an effective first-line therapy for patients with MSI-H mCRC. The data confirm the enduring clinical benefits of using nivolumab and low-dose ipilimumab to treat MSI-CRC patients, suggesting that combining PD-1 inhibitors with other immune therapies can target tumor cells through diverse mechanisms to overcome drug resistance challenges.

Besides the PD-1 pathway, CTLA-4 acts as a crucial immune checkpoint inhibitor that negatively regulates T cell stimulation by binding more strongly to CD80 and CD86 on MHC, thus suppressing self-immune responses (Johdi and Sukor, 2020). CTLA-4 is mainly found in activated CD4^+^ and CD8^+^ T cells. Initially, at low CTLA-4 levels, CD80/86 interactions with CD28 dominate, leading to T cell activation, cytokine secretion (such as IL-2), Bcl-xl production, cell expansion, and differentiation into effector T cells. Subsequently, CTLA-4 competitively binds to CD80/86, outcompeting CD28 and halting the T cell response (Hosseini et al., 2020; Hossen et al., 2023). The absence of CTLA-4 in Treg cells impairs their immunosuppressive function, leading to inappropriate activation and proliferation of conventional T cells (Read et al., 2006). Ipilimumab, the first FDA-approved CTLA-4 blocker for melanoma, is commonly used in CRC treatment as an adjunctive or rescue therapy (Rotte, 2019; Camacho, 2015). The NICHE study demonstrated that administering one dose of ipilimumab along with two doses of nivolumab before surgery resulted in complete pathologic regression in all 20 CRC patients with dMMR, suggesting this dual treatment as a promising standard neoadjuvant approach for dMMR/MSI-H CRC patients (Chalabi et al., 2020). Tremelimumab, another CTLA-4 inhibitor, has been approved for use in combination with durvalumab as the primary therapy for unresectable hepatocellular carcinoma (uHCC) in adults (Keam, 2023). In a study involving advanced colorectal cancer, the combination therapy of tremelimumab and the PD-L1 inhibitor durvalumab improved overall survival (OS) to 6.6 months in the combined treatment cohort, compared to 4.1 months in the monotherapy cohort. Patients with elevated TMB were more likely to benefit from this combined regimen (Chen et al., 2020a). Blocking CTLA-4 reduces the infiltration of MDSCs in the tumor microenvironment (TME), diminishing their immunosuppressive effects and potentially enhancing the efficacy of PD-1/PD-L1 inhibitors in tumor suppression (Rotte, 2019). While MSI-L/MSS CRC remains a significant challenge in CRC treatment, clinical trials like CheckMate 142 and MAYA have shown that combining Nivolumab and ipilimumab, with or without Temozolomide, can lead to disease remission in MSS CRC patients. This supports the potential of using combinations of ICIs with other medications as a treatment strategy for MSS CRC (Overman et al., 2017; Morano et al., 2022). The CO.26 trial (NCT 02870920) assessed the effectiveness of durvalumab combined with tremelimumab versus Best Supportive Care (BSC) in advanced MSS-type CRC. Results indicated a significant increase in OS for the combined therapy group, although PFS did not show significant improvement (Chen et al., 2020b). This suggests that anti-PD-1 and anti-CTLA-4 therapies may prolong survival in MSS-type CRC patients, providing new treatment possibilities. Consequently, further research is necessary to evaluate the feasibility of this combination therapy in MSS-CRC patients to address the challenges posed by these tumors.

### 3.2 LAG3, TIM3, TIGIT, IDO

CTLA-4, LAG3, TIM-3, and PD-1 are the primary co-inhibitory checkpoints involved in tumor development and progression in CRC (Merhi et al., 2023). Although drugs targeting PD-1/PD-L1 and CTLA-4 have shown efficacy in clinical practice, many patients do not respond to checkpoint therapy. The expression of LAG3 on tumor-infiltrating lymphocytes (TILs) in some CRC tissues, where positive expression is associated with advanced tumor staging, MSI-H, and poor prognosis, may serve as a potential prognostic marker for CRC (Xu et al., 2021). Preclinical data and mechanistic analyses suggest that LAG3 could be the third significant checkpoint in clinical applications (Ruffo et al., 2019; Shi et al., 2021; Mariuzza et al., 2024). The combination of the LAG3 inhibitor Relatlimab with Nivolumab is approved for treating metastatic melanoma, marking the debut of FDA-approved LAG3 monoclonal antibody combination therapy. Studies also show that combining Relatlimab with Nivolumab for advanced melanoma treatment significantly improves PFS to 10.1 months compared to 4.6 months with Nivolumab alone (Tawbi et al., 2022). LAG3 contributes to tumor immunity by regulating methylation, influencing the production of immune-related cytokines, and promoting treatment resistance (Shi et al., 2021). A recent study demonstrated that CD8^+^ T cells deficient in both PD-1 and LAG-3 exhibited enhanced tumor clearance and improved long-term survival compared to those lacking either PD-1 or LAG-3 alone. LAG-3 and PD-1 synergistically promote T cell exhaustion and serve as key regulators of TOX expression (Andrews et al., 2024). The research team conducted a clinical trial (NCT03743766) in which advanced melanoma patients were treated with Relatlimab, Nivolumab, or a combination of Relatlimab and Nivolumab to explore the immune mechanisms underlying the combination therapy. The combination of Relatlimab and Nivolumab enhanced CD8^+^ T cell receptor signaling, modified CD8^+^ T cell differentiation, and increased cytotoxicity, while preserving exhaustion characteristics (Cillo et al., 2024). The negative immune regulatory factor T cell immunoglobulin and mucin domain-containing protein 3 (TIM3) acts against tumors. Increased TIM3 expression reduces Th1 cell activity and decreases the secretion of IFN-γ and other anti-tumor factors, thereby weakening the anti-tumor immune response (Pang et al., 2021). Beyond serving as a marker of T cell exhaustion, TIM-3 also plays a critical role in regulating the function of myeloid cells, including macrophages, dendritic cells, neutrophils, and mast cells (Dixon et al., 2024). A study suggests that the inhibitory function of TIM-3 depends on cis- and/or trans-interactions with the adhesion protein CEACAM-1 (Huang et al., 2015). Three additional ligands bind to TIM-3 and regulate antitumor immunity: Galectin-9 interacts with TIM-3 via its glycan chain to modulate TH1 cell immunity through apoptosis; TIM-3 facilitates the clearance of apoptotic bodies in the tumor microenvironment (TME) by interacting with PtdSer; and the HMGB1-TIM-3 interaction disrupts the innate immune response to nucleic acids mediated by Toll-like and cytoplasmic receptors, thereby reducing the effectiveness of DNA vaccines and cytotoxic chemotherapy (Andrews et al., 2019). Indoleamine 2,3-dioxygenase (IDO) plays multiple roles in tumor activation, including inhibiting T cells and NK cells, promoting the activity of Tregs and myeloid-derived suppressor cells, and stimulating tumor angiogenesis (Prendergast et al., 2014; Meireson et al., 2020). The absence of IDO significantly reduces lung vascular density in a mouse model of lung cancer, mainly by decreasing medium- and small-sized vessels, with no effect on large vessels (Smith et al., 2012). IDO1 can catabolize tryptophan into kynurenine, and tryptophan is essential for T cell activation. Studies have demonstrated that the tryptophan-kynurenine (Trp-Kyn) metabolic pathway is linked to local immune suppression within the TME. Increased activity of the Trp-Kyn metabolic pathway depletes tryptophan and leads to the accumulation of metabolites, such as kynurenine, which promotes immune evasion by cancer cells (Hornyák et al., 2018). Epacadostat, an IDO inhibitor, functions to inhibit the degradation of tryptophan, thereby activating T cells to mount an immune response that can suppress or eliminate tumor tissue (Fujiwara et al., 2022). TIGIT is predominantly expressed in activated T cells, Tregs, memory T cells, and NK cells (Ge et al., 2021). TIGIT exerts its immunosuppressive function by binding to CD155 on dendritic cells (DCs), thereby modulating the cytokine production of DCs and indirectly affecting T cells (Joller et al., 2011; Yu et al., 2009). TIGIT induces T cell exhaustion indirectly by competing with the costimulatory receptor CD226 for CD155 binding. Studies have demonstrated that the absence of CD226 impairs T cell antiviral and antitumor responses (Scharf et al., 2020). In chronic viral infections and cancer, TIGIT binds to CD155 with higher affinity, suppressing CD226 and inducing exhaustion in the majority of CD8^+^ T cells (Yu et al., 2009; Stengel et al., 2012; Johnston et al., 2014). Additionally, the concurrent blockade of TIGIT and PD-1 establishes a foundation for specific combination therapies in clinical practice (Banta et al., 2022). In MSI-CRC, immune checkpoint molecules such as IDO1, LAG3, and TIGIT exhibit increased expression compared to MSS-CRC. Combining checkpoint inhibitors appears more effective for CRC with elevated immune cell cytotoxicity (CYT) and microsatellite instability (Zaravinos et al., 2019). Emerging immune checkpoints represent a promising area of study. Although these new drugs are in early development, their potential clinical applications hold significant promise, offering optimism for both the medical field and patients.

## 4 Benefits and biomarkers of immunotherapy

TMB, Combined Positive Score (CPS), and Tumor Proportion Score (TPS) have become pivotal biomarkers in oncology, essential for assessing the suitability of CRC patients for ICI treatment (Klempner et al., 2020). An elevated TMB is associated with a better response to ICIs, indicating a higher probability of the immune system effectively targeting tumor cells (Cao et al., 2022). CPS assesses the ratio of PD-L1 expression across tumor cells, lymphocytes, and macrophages relative to the number of viable tumor cells, providing a holistic view of the tumor microenvironment’s immunogenic potential. In contrast, TPS measures the proportion of viable tumor cells expressing PD-L1, offering detailed insights into tumor cell properties (De Marchi et al., 2021).

Despite extensive research on the correlation between PD-L1 expression and immunotherapy efficacy, its predictive accuracy remains controversial (Havel et al., 2019). The efficacy of ICIs is linked to several biomarkers in CRC, with TMB being particularly notable (Li et al., 2021). TMB serves as a surrogate marker for the neoantigen load within tumors. High TMB tumors present a diverse range of neoantigens to the immune system, thereby enhancing the effectiveness of ICIs in recognizing and eliminating tumor cells (Samstein et al., 2019). A higher TMB generates more neoantigens, increasing the likelihood of T cell-specific recognition. ICI-based cancer immunotherapy can overcome immune evasion by cancer cells (Chan et al., 2019). In colorectal cancer, the correlation between TMB and dMMR or MSI-H subgroups is particularly notable, resulting in elevated TMB levels. The study results indicate that patients with a TMB ≤ 23 mut/Mb have significantly worse PFS and OS. In patients with a TMB > 40 mut/Mb, combination therapy with anti-PD-(L)1 and anti-CTLA-4 monoclonal antibodies significantly outperforms single-agent anti-PD-(L)1 therapy (Manca et al., 2023). TMB has emerged as a key biomarker for predicting the efficacy of immunotherapy in CRC(95). Continued exploration and validation of TMB, along with advancements in genomic analysis, will further enhance the personalization of immunotherapy. Recent studies have highlighted the significance of CPS and TPS in predicting the efficacy of ICIs in CRC. PD-L1-positive tumors typically indicate that tumor cells are more likely to interact with immune cells via the PD-L1/PD-1 signaling pathway, thereby inhibiting immune cell activity and promoting tumor immune escape. Studies have shown that patients with higher PD-L1 positivity in tumors typically exhibit better responses to immunotherapy (Gandhi et al., 2018). Patients with high PD-L1 expression tend to exhibit higher response rates to ICI treatment. For example, in a clinical trial focusing on advanced gastric cancer and gastroesophageal junction cancer, higher PD-L1 expression was associated with improved overall survival rates with pembrolizumab treatment, demonstrating sustained antitumor activity and underscoring the significance of this marker in patient selection (Fuchs et al., 2018; Muro et al., 2016). Similarly, TPS is a crucial predictive factor for the response to PD-1/PD-L1 inhibitors in non-small cell lung cancer (NSCLC). Higher TPS scores are associated with improved treatment outcomes and enhanced responses (Wu et al., 2021). The results of the KEYNOTE-010 study, published in 2016, confirmed the efficacy of a TPS ≥ 50% cutoff for pembrolizumab monotherapy versus docetaxel as a second-line treatment for NSCLC (Herbst et al., 2016). The subsequent KEYNOTE-024 study confirmed the sustained efficacy of a TPS ≥ 50% cutoff for pembrolizumab monotherapy as a first-line treatment in NSCLC patients with PD-L1 TPS ≥ 50%. Pembrolizumab monotherapy exhibited superior efficacy over platinum-based chemotherapy in NSCLC patients with PD-L1 TPS ≥ 50% (Reck et al., 2016). The CPS was first investigated in the context of immunotherapy for advanced head and neck squamous cell carcinoma in studies such as KEYNOTE-012 and KEYNOTE-055 (Mehra et al., 2018; Bauml et al., 2017). The KEYNOTE-012 study demonstrated a significant association between both TPS and CPS and clinical benefit. Patients with CPS-defined PD-L1 positivity showed a higher disease response rate compared to PD-L1-negative patients (21% vs. 6%, *p* = 0.023), along with significant differences in PFS and OS between the groups. The KEYNOTE-055 study indicated that a higher CPS was associated with a greater ORR. The ORR reached 27% for CPS ≥ 50, compared to 18% for CPS ≥ 1. A comparison of TPS and CPS sensitivities using ROC curves revealed that CPS had higher sensitivity, while TPS showed higher specificity in HNSCC.In summary, CPS and TPS play crucial roles in guiding the use of ICIs in CRC. Increasing evidence supports their role in predicting treatment outcomes and facilitating personalized therapy. Furthermore, POLE mutations have predictive value in CRC patients and are associated with increased levels of immune cell infiltration, potentially enhancing tumor sensitivity to immune therapy (Picard et al., 2020). POLE mutations may lead to DNA replication disorders and tumor hypermutation, making patients more likely to have high microsatellite instability. Patients with POLE mutations have a higher 5-year disease-free survival rate compared to those with MSS and MSI-H CRC (Mo et al., 2020). The impact of POLE mutations on immunotherapy extends to their interaction with the tumor microenvironment. These mutations cause alterations that promote tumor cell mutability and enhance immunogenicity, resulting in increased immune cell infiltration (Wang et al., 2023b; Domingo et al., 2016). Clinical studies indicate that tumors with POLE mutations are more responsive to immunotherapy, showing improved outcomes and longer survival. Therefore, a comprehensive evaluation of these biomarkers facilitates more precise assessments of immunotherapy responses and is crucial for devising personalized treatment plans.

## 5 Effects of chemoradiotherapy, targeted therapy combined with immunotherapy on colorectal cancer

### 5.1 Mechanism and progress of immunotherapy combined with chemoradiotherapy

**FIGURE 1:**
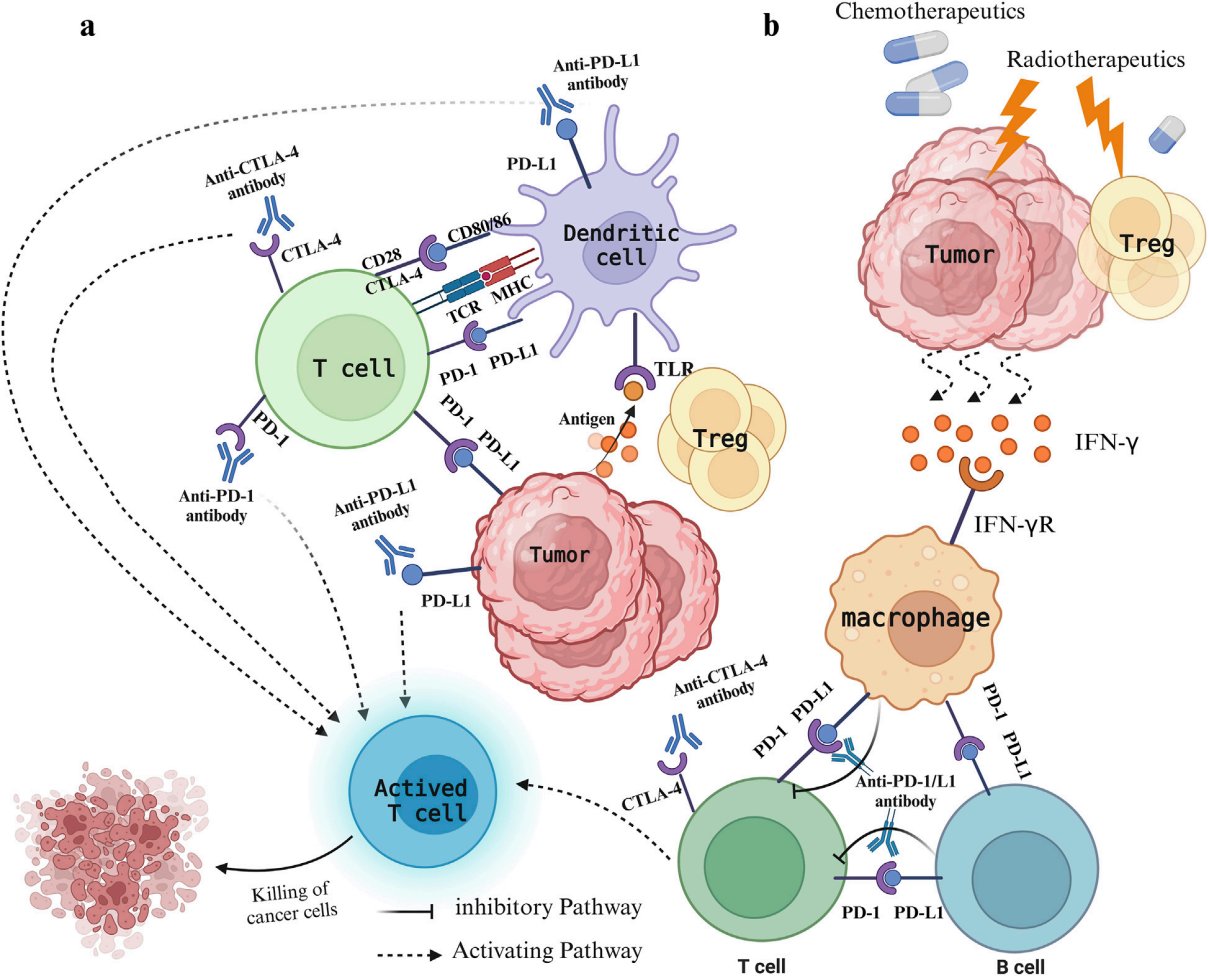
Immune status of patients with colorectal cancer under different treatments. **(A)** Mode of action of PD-1/PD-L1 and CTLA-4 and their inhibitors. The expression of CTLA-4 on T cells enables binding to CD80/CD86 molecules, preventing the CD28^−^CD80/86 interaction, which leads to decreased T cell activation and immune response suppression. PD-1 binds to PD-L1 on either cancer cells or immune cells, resulting in T cell exhaustion and immune system evasion. Inhibitors targeting CTLA-4 and PD-1/PD-L1 prevent their binding to ligands, thereby decreasing negative regulatory signals on T cells, stimulating T cell activation, and bolstering anti-tumor immune responses. **(B)** The leading role of radiotherapy and chemotherapy. Chemotherapy drugs promote immunogenic cell death and impede tumor evasion, whereas radiotherapy causes damage to tumor cells, leading to the release of various damage-associated molecular patterns that activate the immune system against tumors. Meanwhile, radiotherapy reduces Treg cells, triggers the release of cytokines like IFN-γ, and stimulates macrophages to interact with T cells and B cells.

PD-1 and CTLA-4 are crucial immune checkpoints in T cells. PD-1 and PD-L1 function by suppressing the proliferation, cytokine release, and cytotoxic function of immune cells. In contrast, CTLA-4 competes with CD28 for binding to CD80/86, dampening the secondary stimulation signal and thereby impeding T cell activation (Rotte, 2019) (Figure 1). Excessive activation or expression of immune checkpoints in cancer can facilitate malignant growth and spread. Inhibitors of PD-1 and CTLA-4 may produce synergistic effects by concurrently targeting both pathways, resulting in improved treatment responses compared to monotherapy with a single immune checkpoint inhibitor (Zhao et al., 2019). Currently, combining ICIs with chemotherapy has shown improved antitumor effects in various solid tumors, providing a potential therapeutic approach for MSS CRC(48). The primary mechanism of action for most chemotherapy drugs involves direct cytotoxicity, primarily aimed at triggering immunogenic cell death and preventing tumor immune evasion, often without considering their impact on the immune system (Yu et al., 2019). Nevertheless, the synergy between radiotherapy/chemotherapy and immunotherapy has been established, with numerous studies demonstrating the efficacy of combined immunotherapy and radiotherapy/chemotherapy regimens (Ban et al., 2021; Ferrara et al., 2020; Yu et al., 2019; Sharabi et al., 2015). Short-term treatment with oxaliplatin (OXA) increased immune cell infiltration in the MC38 mouse model, enhancing the efficacy of immune checkpoint therapy (Wang et al., 2017). This indicates that combining OXA with PD-1/PD-L1 inhibitors may improve MSS CRC’s responsiveness to immunotherapy. Chemoradiotherapy can significantly reduce tumor volume, decrease the invasion of surrounding tissues and organs, and improve patient survival rates. It has also shown significant progress in perioperative treatment, which is crucial for reducing local recurrence and metastasis (Matzner et al., 2020; Bahadoer et al., 2021). Chemotherapy can eliminate immunosuppressive cells, improve effector cell function, reduce Tregs with low-dose chemotherapy, and shift tumor-associated macrophages from an M2-like to an M1-like phenotype (Yi et al., 2022). The combination of radiotherapy and chemotherapy can inhibit tumor cells and Tregs and stimulate the release of cytokines like INF-γ, TNF-α, and IL. Furthermore, with ICI blockade, CD4^+^ T and CD8^+^ T cells are prompted to produce INF-γ, promoting T cell proliferation and activation (Ramos-Casals et al., 2020) (Figure 1). Reports indicate that oxaliplatin enhances the antigen-presenting capabilities of tumor cells by increasing MHC class I surface expression, thereby facilitating more effective T cell activation and improving ICI-based immunotherapy (Park et al., 2019).

Combining immunotherapy with traditional treatments may enhance the effectiveness of cancer immunotherapy. Growing evidence supports the clinical benefit of pairing appropriate chemotherapy doses with ICIs (Galluzzi et al., 2020). While the outcome of neoadjuvant immunotherapy with chemotherapy remains unclear, short-term chemotherapy has been shown to enhance early immunotherapy efficacy (Paz-Ares et al., 2021). Immunotherapy is generally ineffective for most MSS-CRC, likely due to inadequate immune cell infiltration (Ganesh et al., 2019). In a single-arm, non-randomized phase II trial (NCT03104439), 40 CRC patients initiating treatment had a disease control rate of 25% and an objective response rate of 10%. Following radiotherapy, the DCR rose to 37% and the ORR to 15%, illustrating that radiotherapy can boost the effectiveness of dual ICIs in tumor immunotherapy (Parikh et al., 2021). Radiotherapy can alter tumor cell immunogenicity, amplify CD8^+^ T cell responses, and upregulate PD-L1 expression on tumor and immune cells within the tumor environment, rendering resistant tumors responsive to PD-1/PD-L1 inhibition (Sharabi et al., 2015; Spiotto et al., 2016).Furthermore, chemotherapy can enhance PD-L1 expression on dendritic cells and boost immune cell infiltration (Heinhuis et al., 2019). Hence, combining chemotherapy with immunotherapy could potentially counteract the primary resistance mechanism of MSS mCRC to ICI monotherapy. The efficacy of this combined approach was validated in a phase II clinical trial, which is especially significant for MSS CRC known for its limited responsiveness to ICIs. Furthermore, when combined with radiotherapy, patients’ responses to immunotherapy were improved (Parikh et al., 2021). Studies indicate that combining short-course radiotherapy with chemotherapy and camrelizumab can lead to a higher rate of pCR in patients with locally advanced rectal cancer (LARC) with favorable tolerability (Lin et al., 2021). In a trial (NCT03388190), the effectiveness of the Nordic FLOX protocol involving oxaliplatin was assessed against FLOX plus Nivolumab in treating MSS-mCRC patients. The findings propose that FLOX could potentially transform MSS tumors into an immunogenic phenotype, facilitating responses to ICIs (Meltzer et al., 2022). Furthermore, ongoing studies explore the synergy between immunotherapy and chemotherapy: one trial examines if adjuvant chemotherapy with Avelumab (PD-L1 inhibitor) enhances disease-free survival in stage III dMMR/MSI-H colon cancer patients (Lau et al., 2020). Combining radiation or chemotherapy with immunotherapy has emerged as a prevalent clinical strategy, offering new insights into the safe and effective treatment of MSS CRC. Despite this, clinical research in this area is still limited, and the full potential and safety of such combination therapies need further exploration.

### 5.2 Mechanism and progress of targeted therapy combined with immunotherapy

**FIGURE 2:**
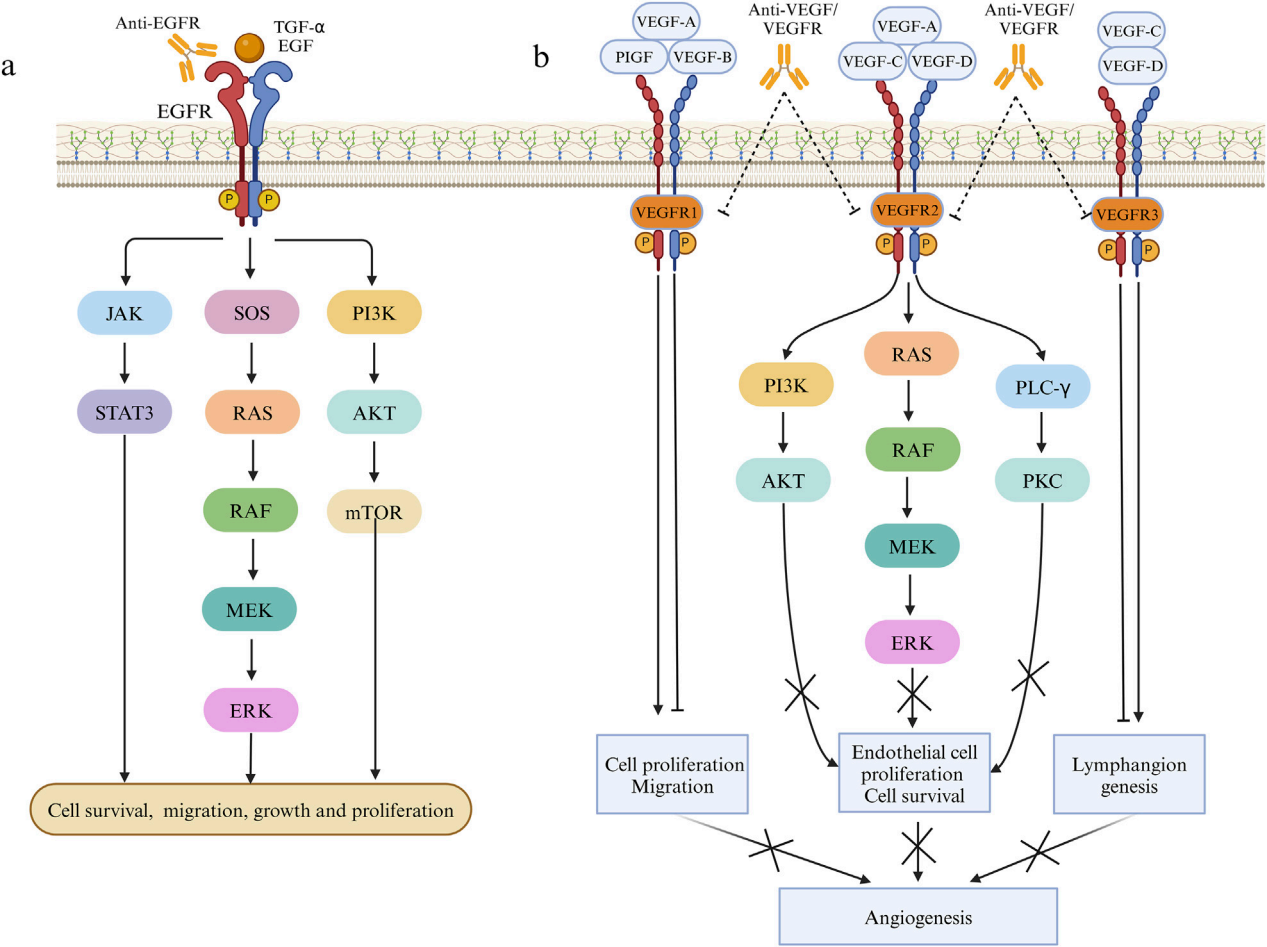
Pathways involved in the mechanism of EGFR and VEGF on cell proliferation and angiogenesis. **(A)** Effects of EGF and TGF-α on cell proliferation, migration and growth through JAK/STAT3, RAS/RAF/MEK/ERK and PI3K/AKT pathways. **(B)** The effect of VEGF family factors combined with VEGFR1/2/3 on angiogenesis.

Radiation therapy and cytotoxic chemotherapy kill replicating cells indiscriminately, targeting both cancerous and healthy tissues. In contrast, targeted drugs offer higher specificity and selectivity, minimizing toxic effects on healthy cells while bolstering treatment outcomes (Karati and Kumar, 2023). Effective targeted therapy necessitates the presence of specific proteins or genetic alterations, hence it commonly targets molecular entities intricately linked to CRC initiation and progression like EGFR and VEGF (Li et al., 2023b) with ongoing exploration of numerous additional targets (Grassilli and Cerrito, 2022). The EGFR is a transmembrane protein of the human EGFR family that can bind to specific ligands like epidermal growth factor and transforming growth factor α. Upon ligand binding, dimerization ensues, triggering the activation of multiple intracellular signaling pathways downstream, involving recruitment of son of sevenless homologs(SOS), RAS/RAF/MEK/ERK, PI3K/AKT/mTOR, and JAK/STAT3, consequently governing cellular processes like growth, survival, and migration (Xie et al., 2020) (Figure 2). Numerous proteins within the EGFR pathway are targeted for CRC treatment. Presently, the FDA has sanctioned several targeted medications targeting pertinent pathways for treating CRC, with ongoing advancements in novel targeted therapeutic approaches (Underwood et al., 2024). The VEGF (now referred to as VEGF-A) family consists of VEGF-A, VEGF-B, VEGF-C, VEGF-D, VEGF-E, and PlGF. The canonical VEGF signaling pathway, mediated by VEGFR-2, modulates kinase function, crucially influencing processes like cell growth, migration, viability, and blood vessel development and angiogenesis (Apte et al., 2019). VEGFR-2, a pivotal signaling molecule, triggers activation of the PLCγ/PKC, Ras/Raf/ERK and PI3K/Akt pathway upon VEGF binding, promoting proliferation of epidermal cells and facilitating neovascularization (Dakowicz et al., 2022). VEGF-A/B binding to VEGFR-1, in conjunction with VEGFR-2, orchestrates angiogenesis, whereas activation of VEGFR-3 through interaction with VEGF-C/D induces lymphatic endothelial cell growth, facilitating lymphangiogenesis (Shaw et al., 2024) (Figure 2). Moreover, the combination of targeted therapy and immunotherapy provides new hope for CRC treatment. Combining targeted therapy with immunotherapy better activates the immune system, leading to improved tumor treatment outcomes. Targeted therapy plays a critical role in modulating the immune environment within the tumor microenvironment. Recent studies by Singh et al. (2024) have thoroughly explored the effects of targeted therapy on the immune microenvironment and the combination of targeted therapy with immunotherapy, offering a more precise and effective approach to CRC treatment. Doleschel et al. (2021) reported that in syngeneic mouse models of MSS CT26 and hypermutated MC38 colorectal cancer, the combined use of regorafenib and anti-PD1 significantly boosted antitumor activity. This therapy also substantially decreased the infiltration of immunosuppressive macrophages and Treg cells into the tumor microenvironment and raised intratumoral IFNγ levels. The trial revealed that regorafenib combined with anti-PD-1 significantly enhances the tumor microenvironment and sustains effective treatment in MSS CRC, demonstrating a synergistic effect. In the multicenter randomized trial TAILOR, the comparison of FOLFOX with or without cetuximab in treating KRAS wild-type CRC revealed enhanced PFS (9.2 months vs. 7.2 months) and OS (20.7 months vs. 17.8 months) in patients administered FOLFOX and cetuximab (Qin et al., 2018). Additionally, another Phase II randomized clinical trial highlighted the substantial benefit of cetuximab combination therapy on PFS and OS in individuals with RAS wild-type mCRC (Boige et al., 2023). The PANAMA trial demonstrated that maintenance therapy with fluorouracil and folinic acid (FU/FA) plus panitumumab significantly enhances progression-free survival (PFS) (8.8 months vs. 5.7 months) and overall survival (OS) (28.7 months vs. 25.7 months) in RAS wild-type mCRC compared to FU/FA alone (Modest et al., 2022). Combining targeted therapy and immunotherapy offers extended survival benefits, and an ongoing Phase II study explores pembrolizumab alone or in combination with BRAF and EGFR inhibitors for patients with BRAF V600E mutation and MSI-H mCRC (Elez et al., 2023). While the preliminary results of immune combined targeted therapy show promise, it is essential to recognize that each patient’s situation is unique. Consequently, there is a need for further individualized and precision medical approaches to determine the most suitable treatment plan.

### 5.3 Dual effects of combination therapy and its limitations

Immunotherapy combined with surgery, radiotherapy, chemotherapy, targeted therapy, or other immunotherapies has emerged as a research focus in recent years; however, such approaches may also act as a double-edged sword (Sarkar et al., 2024). Chemotherapy enhances the immunogenicity of tumor cells, overcomes immune suppression, and sensitizes tumor cells to immune attacks. In contrast, immunotherapy has the potential to eradicate disseminated disease by leveraging the body’s immune system (Luo et al., 2019). Radiotherapy stimulates immune cells to recognize cancer cells and enhances the immunogenicity of colorectal tumors, thereby augmenting the efficacy of ICIs (Shi et al., 2022; Seyedin et al., 2020). Radiotherapy enhances the host immune system’s ability to recognize and eliminate tumor cells by upregulating MHC class I molecule expression on tumor cell surfaces, increasing CD8^+^ and CD4^+^ T lymphocyte infiltration, and improving tumor cell antigen recognition (Jin et al., 2023; Zeng et al., 2019). However, radiotherapy also has certain negative immunosuppressive effects. This leads to the accumulation of double-stranded DNA (dsDNA) within tumor cells, activating the cGAS/STING signaling pathway and promoting type I interferon gene transcription (Long et al., 2023). In certain cases, interferon signaling may have detrimental effects. For instance, repeated irradiation of tumor cells can induce chronic type I interferon and interferon-stimulated gene expression, driving treatment resistance and tumor immune evasion via various inhibitory pathways (Boukhaled et al., 2021; Kumar et al., 2023). Studies have shown that radiotherapy not only upregulates PD-L1 expression on tumor cell surfaces but also modulates the expression of various immune checkpoint ligands, including PD-L1, on immune cells within the tumor microenvironment, generating an immunosuppressive antitumor effect (Du et al., 2022; Wu et al., 2023). Furthermore, the immune response induced by radiotherapy may depend on the radiation dose. Studies have shown that low-dose rate irradiation can remodel the tumor immune microenvironment (TIME), enhancing the therapeutic response to ICIs and improving the infiltration and function of effector immune cells in distant tumor sites (Patel et al., 2021; Barsoumian et al., 2020). As two synergistic treatments, immunotherapy also enhances the efficacy of radiotherapy. Studies have shown that ICIs not only activate cytotoxic T cells but also normalize tumor blood vessels, alleviate hypoxia, and increase tumor sensitivity to radiotherapy (Tian et al., 2017; Zheng et al., 2018). Immunotherapy can deplete Treg cells and modulate the phenotypic states of tumor cells, immune cells, and mesenchymal cells, thereby improving their sensitivity to radiotherapy (Du et al., 2022). Growing evidence suggests that the immune system plays a crucial role in the response to radiotherapy and chemotherapy. The combination of radiotherapy, chemotherapy, and immunotherapy acts synergistically, but it also has limitations and may cause severe side effects. The most common side effects of combining anti-PD-1 or anti-PD-L1 antibodies with chemotherapy, targeted therapy, immunotherapy, and radiotherapy are anemia (45.4%), fatigue (34.3%), and dysphagia (30.0%) (Zhou et al., 2021). Some meta-analyses indicate that the incidence of treatment-related toxic side effects is significantly higher with combination therapy of anti-PD-1 or anti-PD-L1 antibodies and anti-CTLA-4 antibodies, compared to monotherapy (Xu et al., 2018; Almutairi et al., 2020). Additionally, combining immunotherapy with targeted therapy (e.g., VEGF or VEGFR inhibitors) is more likely to cause hypertension and severe proteinuria, with these rare adverse events linked to the anti-angiogenic effects of the drugs (Wu et al., 2010; An et al., 2010). In the chemotherapy-immunotherapy combination regimen, the incidence of treatment-related adverse events reaches 97.7%, with 35.9% classified as grade 3 or higher (Zhou et al., 2021). Additionally, radiotherapy combined with ICIs seems to be better tolerated than when combined with targeted therapies or chemotherapy drugs (Kroeze et al., 2017). Therefore, when addressing different tumor types and developing combination regimens, the mechanisms of action of various ICIs should be considered to minimize severe adverse reactions in patients.

## 6 Discussion and prospect

In the last 2 decades, there has been a notable surge in interest and research on targeted therapies for CRC, resulting in several promising new therapies approved by the FDA for treating mCRC. Compared to MSS tumors, the tumor microenvironment of dMMR/MSI-H CRC shows a significant upregulation of immune checkpoints like PD-L1, CTLA-4, LAG-3, and IDO, which generate tumor-associated immunogenic antigens (Le et al., 2015; Toh et al., 2016). These observations have highlighted the feasibility of checkpoint blockade in MSI-H CRC treatment. Simultaneously, the rapid advancement of ICIs has led to their approval for CRC treatment. Despite the significant efficacy demonstrated by CTLA-4 and PD-1/PD-L1 inhibitors, the overall effectiveness of current ICI therapy remains modest due to the complex tumor microenvironment and tumor heterogeneity. Thus, enhancing tumor response to immunotherapy is a primary research goal, emphasizing the development of combination therapies that utilize various ICIs in conjunction with radiotherapy, chemotherapy, and targeted therapy. MSS CRC, characterized as a ‘cold tumor’ resistant to ICIs, presents significant immunotherapy challenges (Lizardo et al., 2020). Nonetheless, mounting evidence indicates that multi-pathway combination therapy with PD-1/PD-L1 inhibitors is clinically promising and merits further research in this context (Li et al., 2023c). In fact, some patients exhibit positive responses to targeted therapy; however, mutations in KRAS and BRAF can cause resistance to specific targeted treatments (Formica et al., 2022). Hence, integrating immunotherapy with targeted therapy could offer a novel strategy for overcoming resistance mechanisms. Moreover, novel biomarkers are required to determine the patients who would derive the greatest benefit from targeted medications (Manzi et al., 2023). Ongoing clinical studies indicate that utilizing these ICIs in isolation may not be adequate for generating strong anti-tumor effects; strategic combinations of immunotherapies can enhance the synergistic effects of anti-tumor immunity.

Chemoradiotherapy is frequently used in treating CRC. Combining immunotherapy with this approach represents an emerging treatment strategy, offering renewed optimism for advanced colorectal cancer patients. Recently, the growing utilization of ICIs combined with chemoradiotherapy has enhanced processes such as the heightened release of tumor antigens, improved antigen presentation, and activation of immune cells. This synergy boosts the immune system’s assault on cancer cells, enhancing treatment effectiveness. Nonetheless, challenges persist, including variable treatment responses, resistance development, and potential toxicities linked to treatment. Consequently, additional research is imperative to refine treatment protocols and advance the development of more potent ICIs and targeted medications. Future research should explore the genomics and immunomics of patients to enhance understanding of the molecular characteristics and immune status of tumors. This will enable the development of targeted therapies that can improve the prognosis of CRC patients. Cancer immunotherapy has advanced significantly, leading to improved survival rates among late-stage patients. Understanding the role of immune checkpoints in the tumor microenvironment is crucial for laying the foundation for the development of ICIs. The application of ICIs has addressed therapeutic obstacles in certain treatment-resistant CRC. Besides established inhibitors like CTLA-4, PD-1/L1, and LAG-3, numerous novel drugs are in development, including TIGIT and TIM-3 inhibitors. This article primarily focuses on the advancements in combining immunotherapy with targeted therapy, chemotherapy, and radiotherapy, all demonstrating specific advantages. Nevertheless, extensive randomized controlled trials are necessary to confirm the efficacy of combination therapy and determine the most effective combinations of immunotherapy for maintaining long-term anti-tumor immune responses.
